# Exploratory mapping of tumor associated macrophage nanoparticle article abstracts using an eLDA topic modeling machine learning approach

**DOI:** 10.1371/journal.pone.0304505

**Published:** 2024-06-18

**Authors:** Chloe Brown, Colette S. M. Bilynsky, Melanie Gainey, Sarah Young, John Kitchin, Elizabeth C. Wayne

**Affiliations:** 1 Department of Chemical Engineering, Carnegie Mellon University, Pittsburgh, Pennsylvania, United States of America; 2 Department of Biomedical Engineering, Carnegie Mellon University, Pittsburgh, Pennsylvania, United States of America; 3 Carnegie Mellon University Libraries, Carnegie Mellon University, Pittsburgh, Pennsylvania, United States of America; 4 Department of Bioengineering, University of Washington, Seattle, Washington, United States of America; Xijing Hospital, Air Force Medical University, CHINA

## Abstract

The role of macrophages in regulating the tumor microenvironment has spurned the exponential generation of nanoparticle targeting technologies. With the large amount of literature and the speed at which it is generated it is difficult to remain current with the most up-to-date literature. In this study we performed a topic modeling analysis of 854 abstracts of peer-reviewed literature for the most common usages of nanoparticle targeting of tumor associated macrophages (TAMs) in solid tumors. The data spans 20 years of literature, providing a broad perspective of the nanoparticle strategies. Our topic model found 6 distinct topics: Immune and TAMs, Nanoparticles, Imaging, Gene Delivery and Exosomes, Vaccines, and Multi-modal Therapies. We also found distinct nanoparticle usage, tumor types, and therapeutic trends across these topics. Moreover, we established that the topic model could be used to assign new papers into the existing topics, thereby creating a Living Review. This type of “birds-eye-view” analysis provides a useful assessment tool for exploring new and emerging themes within a large field.

## Introduction

Macrophage nanoparticle targeting in cancer immunotherapy is an exploding field. Tumor-associated macrophages (TAMs) are a prime target for biologists who seek to understand their role in tumor progression and immune evasion. Lixkewise, they have been a target for engineers who design therapeutics to reprogram TAM functions. The enormous amount of literature generated spans multiple scientific disciplines, institutions, and geographical regions. Moreover, differences in disciplines can lead to different nomenclatures, which may limit the accuracy of traditional literature search strategies. In addition, literature is accessed across numerous databases. These complexities can make it difficult for any researcher to stay current.

A scoping review is a powerful tool that gives researchers a broad picture of a field by extracting data from literature related to a specific research question. To accomplish this goal, papers are processed through multiple searching, screening, and data extraction phases. Researchers can then review an entire volume of literature and obtain both a qualitative and quantitative view of a field. This systematic approach has other advantages as well. It can reduce bias that is introduced when reviewing a smaller number of papers. Furthermore, utilizing a protocol for screening papers can help identify emerging research trends.

One of the challenges of a scoping review is the time and work needed to screen and extract data from thousands of papers. Machine learning, specifically natural language processing, has been used to aid in multiple phases of a scoping review [1–3]. Previously, topic modeling, a form of unsupervised machine learning, was used to create an overview of the types of papers in a literature review based on their abstracts [3, 4]. These include but are not limited to cancer immunotherapy [5], emergency medicine [6], and HIV-AIDS research [7]. While these types of analyses cannot replace the level of detail obtained by time-consuming manual data extraction, they provide an overview of these literature datasets that are too large to analyze feasibly manually.

In this paper, we generate a topic model of literature about macrophage targeting cancer therapy using the Ensemble Latent Dirichlet Algorithm [8, 9]. The articles used in this project resulted from a full-text screening process using pre-defined eligibility criteria, previously described in “Scoping Review of Pre-clinical and Translational Studies on Macrophage Polarization in Nanoparticle–based Cancer Immunotherapy” [10]. Additionally, using this model, we demonstrate how topic models can be used to create a “living scoping review” where existing topics are extracted from new articles. This type of meta-analysis provides a valuable assessment tool for summarizing abstracts. It is a useful tool for any user wishing to gain a global summary of a new topic before performing in-depth analysis.

## Materials and methods

### Datasets and code

The datasets used to create this model can be found in the supplemental information. Additionally, a Jupyter notebook containing sample code to create the model and figures can be found in the supplemental material. Finally, the files that contain the model can be found in the supplemental material.

### Obtaining the dataset

The dataset for this review was obtained using guidance from several scoping review methodology resources [11]. Scoping reviews are used to obtain a near-comprehensive overview of a particular body of literature with a well-defined scope. To minimize selection bias, a systematic approach to searching for and identifying relevant articles is used. With this approach in mind, a protocol was developed and registered on the Open Science Framework [10]. A search string was crafted by two of the authors, who are information specialists with experience in evidence synthesis methods [MG, SY] to search for articles containing information about nanoparticles and macrophage polarization in a cancer context [S1 File]. We searched the following bibliographic databases: Web of Science Core Collection [including Science Citation Index-Expanded, Social Science Citation Index and Emerging Sources Citation Index], Scopus, IEEE Xplore Digital Library, Medline (PubMed), and Biotechnology & BioEngineering Abstracts (ProQuest). Hand-searching journals, references from related literature reviews, and Google Scholar found additional articles. The database searches were run between April 23, 2020 and October 20, 2020. We chose only to include articles published in 2000 or later due to the large growth of research in cancer nanotherapeutics in the last twenty years. Due to the large number of included articles and limited resources, we did not conduct forward or backward citation searching.

The articles, hereafter referred to as records, underwent the first few stages of a scoping review, including de-duplication, title and abstract screening, and full-text screening [12]. In the de-duplication phase, records that appeared multiple times in the dataset were consolidated into one entry. This work was done in Zotero.

In the title-abstract screening phase, records were labeled as “include” or “exclude” based on a set of pre-defined criteria [10]. These criteria included whether the study was carried out in a cancer context, included at least one nanoparticles characterization identifier, and contained at least one measurement for macrophage polarization. The heterogeneity in how researchers report these features necessitated flexibility in paper criteria that would allow reviewers to use their judgment at the full text level about paper acceptance into the study. For example, while nearly every paper cited size and composition not every study included encapsulation efficiency, charge, stability etc., For groups with established nanoparticle technologies, it was also a common practice to reference previous references for additional characterization information. The use of two independent reviewers and a third expert reviewer when there was a conflict affirmed the rigor of our screening process and that the manuscripts in our topic model fit were accurately included.

Records were again labeled “include” and “exclude” during the full-text screening, based on more refined criteria. As with the previous screening stage, inclusion decisions needed to be consistent between two independent reviewers, with conflicts resolved by a third expert reviewer. The excluded papers in this section were also labeled with an exclusion reason. The title-abstract and full text screening phases were carried out in Sysrev [13].

We used Sysrev’s built-in machine learning capabilities to automatically exclude articles in our large dataset [13]. We first trained the Sysrev inclusion prediction algorithm by manually screening half (7,482) of the records in the initial title-abstract screening phase. We then used its prediction to automatically exclude records with a prediction value of less than 40% (3,460 records). We found that 14 of the 7,482 articles (0.19%) in the training dataset had been incorrectly classified for exclusion by the prediction algorithm when using a less than 40% prediction value. These records were manually included in the full-text screening phase of the project. Records identified from Google Scholar, Biotechnology & BioEngineering Abstracts (ProQuest), and hand searching were not used to train the algorithm. They were manually screened for inclusion at a later stage of the project.

The topic model used in this study was created using 854 of the abstracts of papers included after the full-text screening (S2 File). An additional 95 abstracts were retrieved using the same search engine strategies to create a living scoping review.

### Data pre-processing

The dataset underwent multiple pre-processing phases, including converting all words to lowercase, tokenization, removing stop words, and stemming.

#### Tokenization

Before the document set was tokenized, they were converted to all lowercase. This was done because the later methods used would be case-specific. Thus, terms such as “Cancer” and “cancer” would otherwise be interpreted as separate words by the computer. The NLTK RegEx tokenizer was used to tokenize the document set [14]. We used the regular expression r’\w+’, which matches all Unicode word characters. In other words, the tokenizer would separate all sequences of alpha-numeric words of length 1 or longer. The tokenizer would not recognize non-alpha-numeric symbols, such as white spaces. After tokenization, each document was converted to a list of tokens. The tokens for each document were then converted to lowercase.

#### Stop words

A list of stop words was created in multiple steps to remove from the data set to isolate words that were more characteristic of a study or topic in particular rather than common of the entire records dataset. Stop word removal is an important data-cleaning step when creating topic models of a set of documents. Stop words are terms that do not provide any meaning or differentiation between the documents. For example, terms such as “the”, “a”, and “and” are considered stop words because they are common terms but do not have significant meaning independently. First, a list of common English stop words was imported from NLTK [14]. Sometimes, a domain-specific list of stop words is needed, such as when an abstract set has a narrow scope [3].

Given the thorough screening process the document set went through before model creation, the documents had concepts that were inherently similar. Thus, we opted to expand on our list of stop words. Next, a list of stop words was created based on the search string. These included words like “cancer”, “nanoparticle”, and “macrophage,” which were common in the dataset, even though they are not common in the English language. Additionally, tokens of length 1, such as “b”, were removed. Finally, words that occurred in once or in 90% of abstracts were removed.

### Stemming

The abstracts were stemmed using the NLTK Porter Stemmer [14]. Words in the abstracts were stemmed by removing word affixes, such as “-ing”, “-ed”, and “de-”, and otherwise isolated words that were between two spaces. We chose to stem the abstracts rather than lemmatize them because many words in the dataset are uncommon in everyday English. Therefore, lemmatization would not work as well on this dataset.

### LDA algorithms

This study used the ensemble latent dirichlet allocation (eLDA) topic modeling algorithm to summarize a large corpus of biomedical text. The traditional LDA model generated noisy, incoherent topics and this result was insensitive to hyperparameter tuning. Therefore, we attempted to use a modified topic modeling algorithm that was developed to decrease the noise associated with the random allocation of topics when initializing LDA [9]. Gensim Ensemble Latent Dirichlet Allocation (eLDA) uses a variation of the DBSCAN algorithm on a collection of LDA topics to distinguish between stable and noisy topics [9]. We found that eLDA had superior performance to the traditional LDA model and used this technique for the scoping review [15]. In addition, we have made the code available for public use [S3 File].

The Gensim eLDA module requires the parameters corpus, consisting of a bag of words model of the documents set, and id2word. The corpus is then mapped to an integer identity. The dictionary was created using the Dictionary module from the Gensim corpora module. The bag of words model of the document set, the corpus, was created by applying the doc2bow function from the dictionary to every abstract in the cleaned dataset.

### Evaluating the topic model

The topic model was evaluated both quantitatively and qualitatively. The model was evaluated quantitatively using the metric average topic coherence, which was measured using the Gensim Coherence Model module [15]. Topic coherence is a measurement used to determine whether the word distribution for a topic is meaningful. A topic that has a high coherence score should have a well-defined theme. A topic with a low coherence score is likelier to be composed of unrelated words. We used the Cv topic coherence, which correlated the best with human measurements in comparison with other topic coherence measurements [16]. This measurement is based on word co-occurrence within sliding-window sections of the documents. Each topic’s coherence measures were averaged to find the average topic coherence, which was used as a metric to evaluate the topic model quantitatively.

The topics created by the models were also evaluated qualitatively for coherence with individuals with domain knowledge in the field. This was done by evaluating the top 30 highest weighted words from each topic distribution as defined by the LDA model.

Additionally, we looked at the 100 most frequent terms to look for trends within each topic based on previous domain knowledge. We defined a record as “belonging” to a topic if that topic had a weight greater than or equal to 0.4 in the topic distribution. Next, the 100 most frequent terms were extracted from the entire set of documents representing that topic. These terms were sorted into categories defined by subject-area experts. These classifications were then compared to their frequency in each of the topics. It is important to note that LDA (and eLDA by extension) produces a weighted distribution of topics for each document and as well as words for each topic. A document can contain more than one topic, and a word can be found in more than one topic.

### eLDA for a living review

We used the 95 new abstracts to demonstrate a “proof-of-concept” for a living review. To do this, we pre-processed the abstracts described above and created a “bag-of-words” model of each document. We then used the eLDA model to extract topics for each model. LDA, and by extension eLDA, can extract topics from documents not previously seen in the dataset, a strategy previously used in classification studies [4, 17, 18]. In this instance, we did not update the model to incorporate the new documents.

## Results and discussion

### Overview of the scoping review and the generated dataset

**Fig 1:**
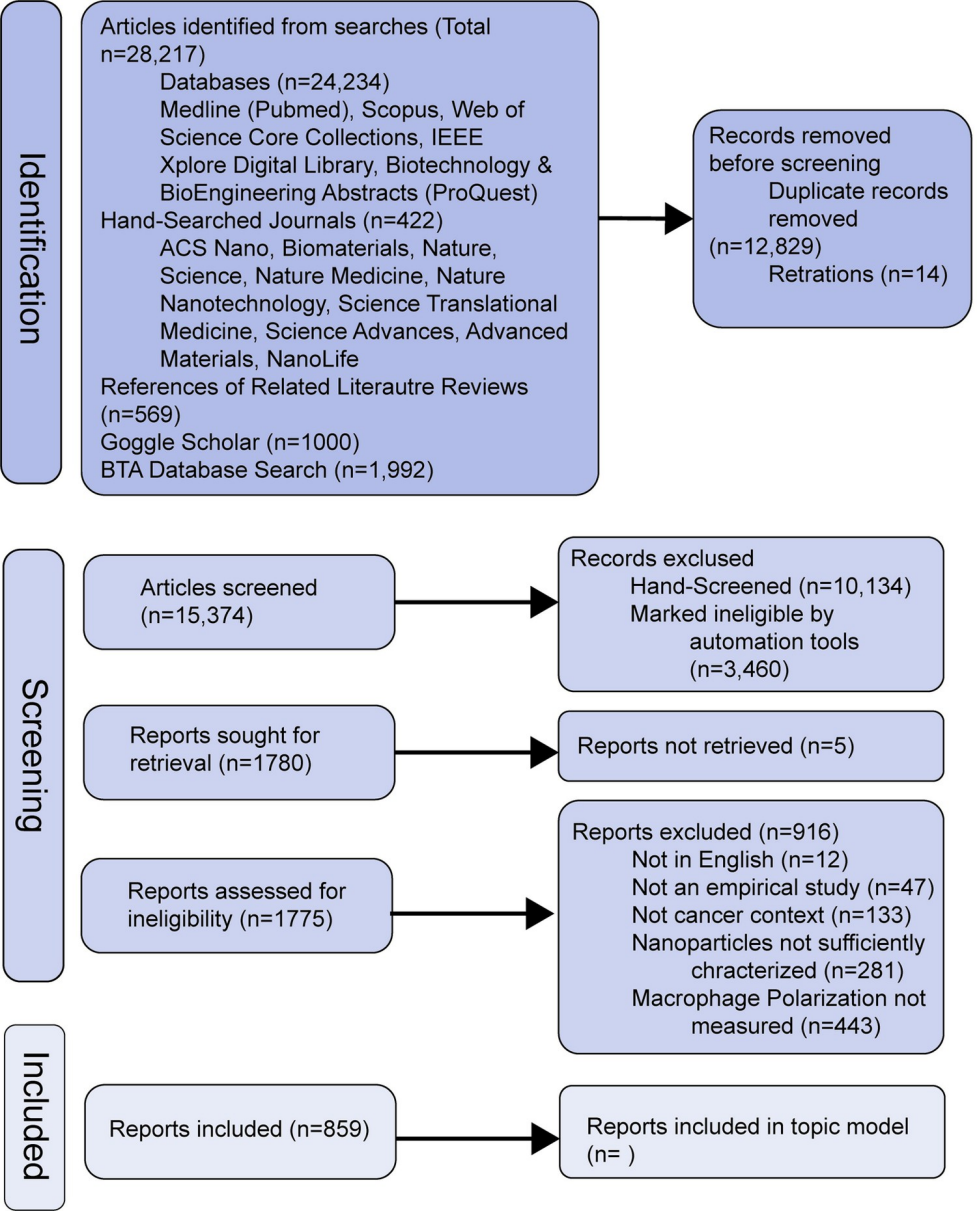
Obtaining the dataset diagram of the scoping review methodology. Flow chart outlining the number of records from article identification, screening, and inclusion into the final data set used in the eLDA topic model. Of the 28,217 articles identified, 854 were included in the topic model that met all screening criteria. 95 newly identified records were used in the Living Review model. Adapted from [19].

The searches of bibliographic databases, handsearching and Google Scholar resulted in 28,217 records. After deduplication and removing retracted records, 15,374 were screened with 854 records included in the final dataset (Fig 1). 854 records were used to train the topic model and 95 new records were used to generate a living scoping review. Note that the 95 new records did not undergo the screening process.

**Fig 2:**
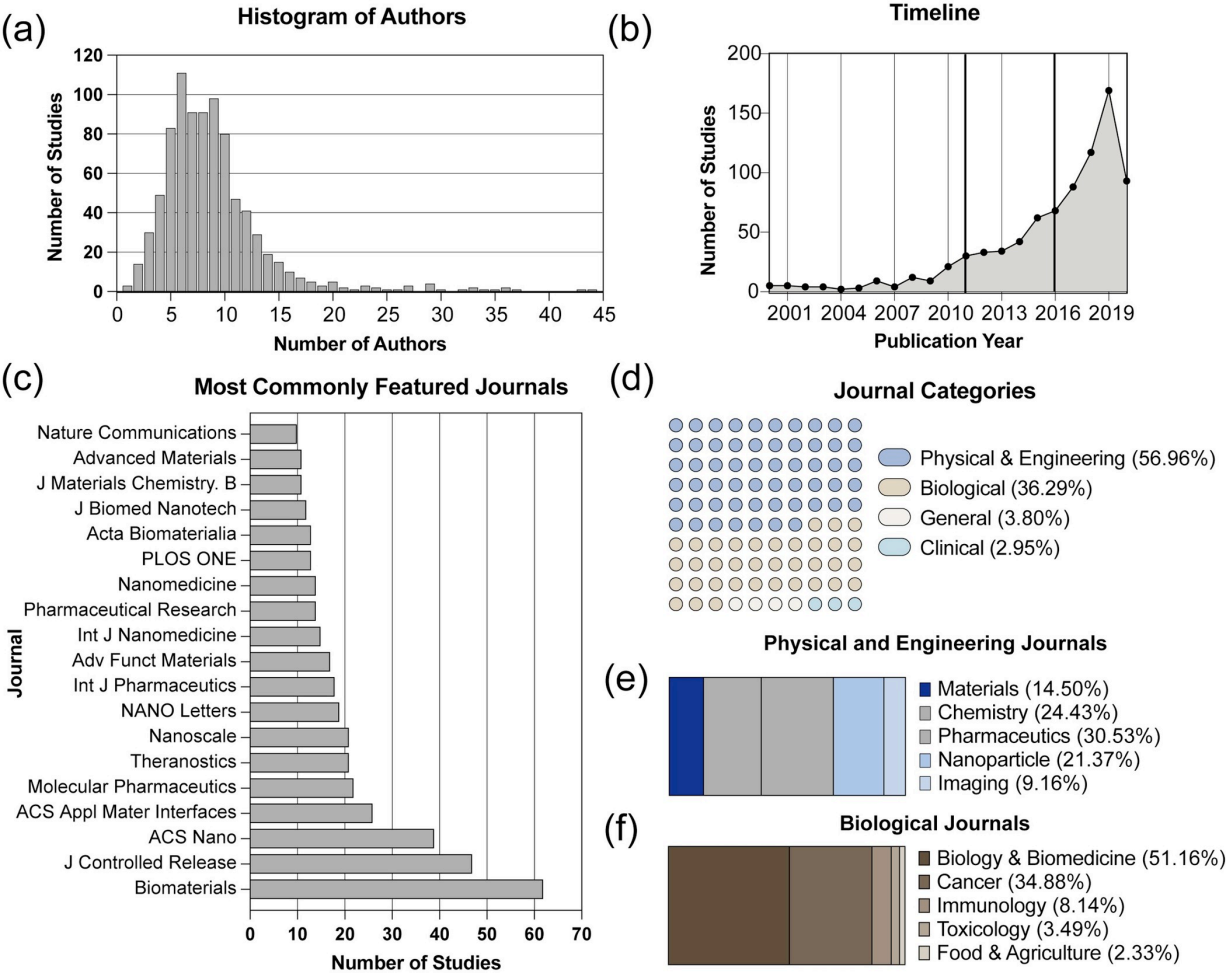
Characterizing the dataset. [a] Distribution of number of authors in the dataset. [b] Number of documents in the dataset over time. Gray lines indicate important advancements in the field. [c] Most common journals in the dataset. [d] Distribution of journal topics included in the dataset. [e] Breakdown of the journal scopes within [e] physical and engineering journals and [f] biological journals.

Before creating the topic model, that dataset was characterized to provide a global view of the included documents **(Fig 2)**. First, we investigated the trends over time in the publications (Fig 2A). We observed two inflection points of growth in the field of macrophage cancer immunotherapy. These represent two growth fields in the immunotherapy field. In 2011 Ipilimumab, the immune checkpoint inhibitor, was approved by the FDA for use in melanoma [20].

Moreover, the 10-year sequel to the original Hallmarks of Cancer included the immune system’s role as a principal biological component in cancer progression and treatment [21]. In 2017, the FDA approved the first CAR T therapy [22]. During this period, it became increasingly apparent that the immune system played a crucial role in finding effective cancer treatments. Both advances utilize T-cells, which is why they have been most useful for tumors with high T-cell invasion and liquid tumors [20]. Macrophages comprise a large portion of the tumor volume and contribute to the immunosuppressive environment that prevents T-cell infiltration [23]. Perhaps for this reason, macrophages specifically became a target for cancer nanotherapeutic research, resulting in exponential growth in this field.

Further bibliographic analysis demonstrated other publishing trends within the field. The median number of authors was about 8, suggesting that this type of research frequently requires a larger collaborative team (Fig 2B). We did not extract data related to author, gender, and institution because the sociological expertise needed to extract and analyze these features properly was viewed to be out of scope. Overall, the articles were published in various journals with Biomaterials, Journal of Controlled Release, and ACS Nano being the most represented (Fig 2C). All three of these journals emphasize the multidisciplinary nature of their publication: physics, chemistry, and biology.

### Characterization of the topic model

**Fig 3:**
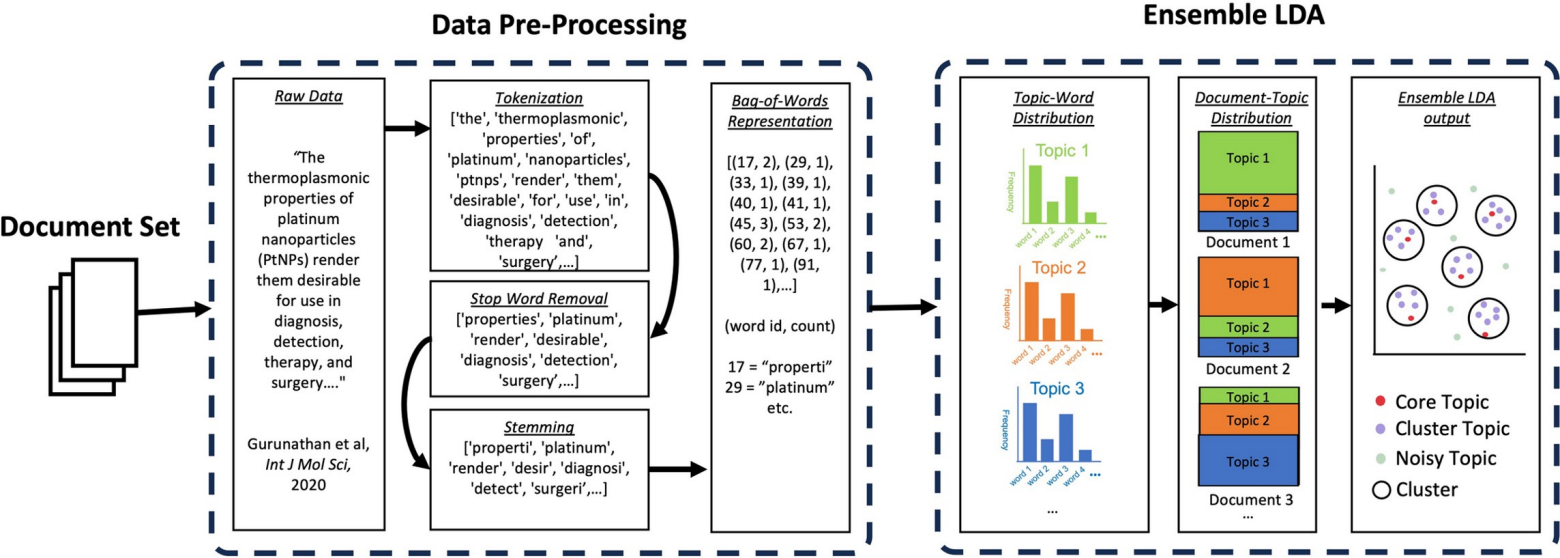
Workflow for creation of eLDA topic model. The document set first underwent multiple stages of preprocessing. This included tokenization, in which documents were divided into a list of tokens, stop word removal, in which common words were removed, and stemming, in which word suffixes were removed. The topic model was created using the ensemble LDA algorithm [4, 16]. This algorithm includes the creation of multiple topic models [4]. The topics are then clustered using a version of the DBSCAN algorithm to differentiate between stable and noisy topics [16]. This was done using the Gensim ensembleLDA module [15].

We used the unsupervised eLDA algorithm to create a topic model of 20 years of literature discussing macrophage polarization in nanoparticle-based cancer immunotherapy [9]. We found six topics that can be used to summarize the corpus. Each document in the corpus is represented as a distribution of topics, and each topic is represented as a distribution of words **(Fig 3)** [4]. Since the model is unsupervised, we did not assign any topics to documents, or words to topics, by hand. However, we found that the topics identified by the topic model correspond to expert-identified trends in the field.

**Fig 4:**
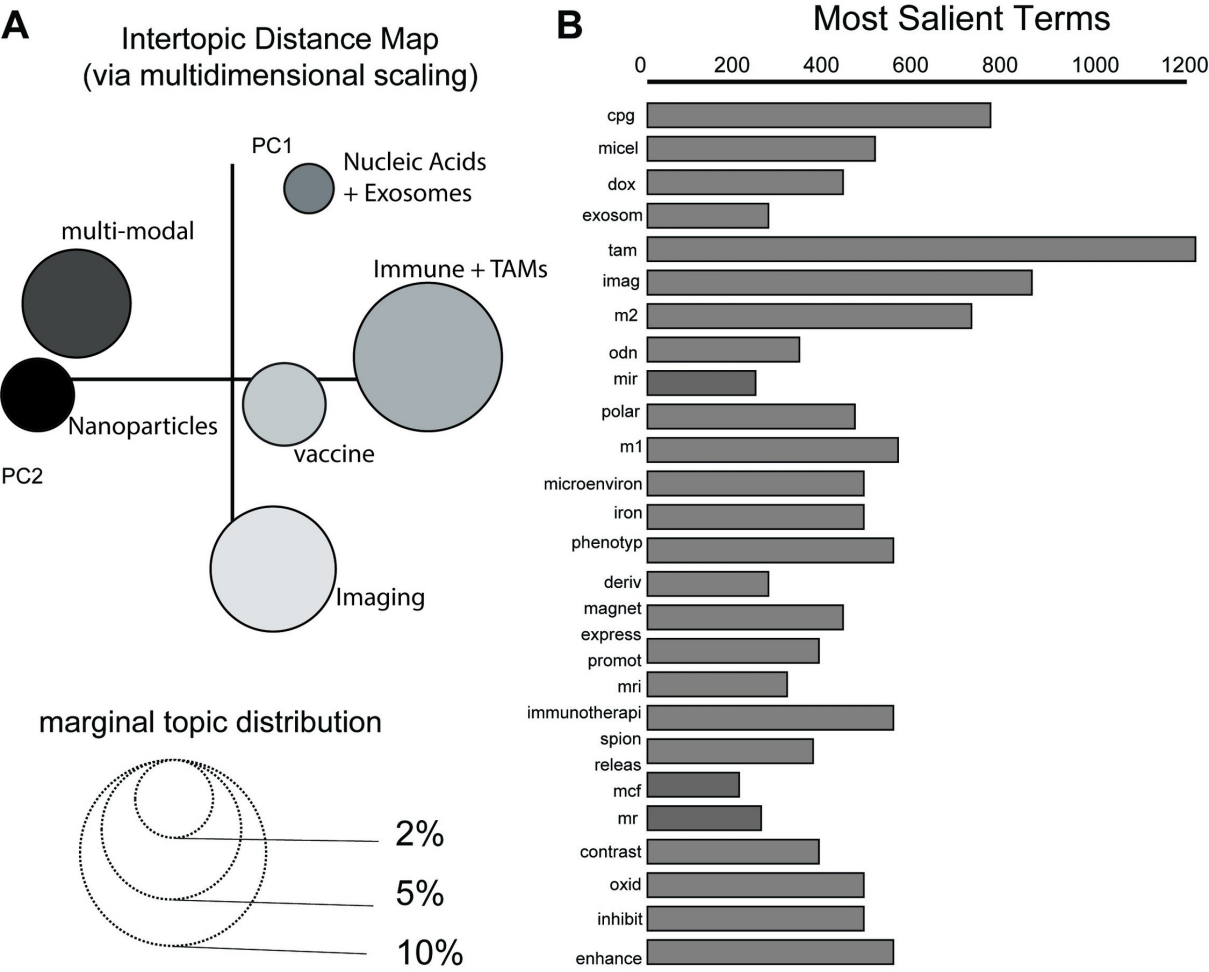
Creating the ensemble LDA topic model. [A] Intertopic Distance Map of LDA topics. Size of the circles represents the relative number of articles within each topic while the distance between circles represents similarity between topics. Marginal topic distribution for each topic indicates the percentage of the dataset that belong to each topic. [B] Most Salient Terms. These are the thirty most frequent terms used to create the topic model. This figure was generated using the pyLDAvis library for topic model visualization in Python [25].

The unsupervised topic model was evaluated both qualitatively and quantitatively to evaluate the model’s accuracy. Average topic coherence, which is the average of the topic coherence scores of each topic, was calculated. The LDA hyperparameters and number of topics are typically tuned using the coherence score. However, we found that varying these hyperparameters did not significantly impact the coherence score. Instead, we chose hyperparameters to create a model that fit our purposes. The Cv coherence score for this model was 0.589. A visualization of the topic model can be found in **(Fig 4)**, which was created using pyLDAvis [25].

For the qualitative evaluation, the topic model was determined to be successful if it produced topics that made human sense. A successful topic model had topics with distinct themes based on the top words and documents assigned to them. The topics were named in accordance with these themes. The topics were qualitatively assigned names based on abstract contents as follows: Immune and TAMs, Nanoparticles, Imaging, Biologics Delivery, Vaccines, and Multi-modal Therapies.

Although the eLDA algorithm effectively reduced the generation of incoherent topics, it did not result in reproducible topics over multiple runs [24, 25]. This is because the LDA algorithm which is the basis for eLDA uses a random allocation to initially assign topics. The random allocation in the model can return different topics when given the same parameters. When utilizing this strategy, we compiled the model multiple times to determine the most persistent topics to represent the dataset. We calculated the average topic coherence for each topic model created and found that it did not vary significantly between runs.

Qualitatively, we found the model most frequently returned to Immunology and Nanoparticle Topics. The remaining topics appeared throughout the model simulations but not as consistently. Imaging and Multi-model Therapies were usually found in the Nanoparticle Topic while, Vaccine, Biologics Delivery topics were contained within the Immunology Topic. This seemed reasonable given the topic proximity on the Intertopic Distance Map (Fig 4A). When evaluating the topic models qualitatively, the models produced using eLDA still make more “human sense” than those created with LDA alone with this dataset. Therefore, while the eLDA algorithm did not create entirely reproducible models, it reliably created meaningful topics. Therefore, the eLDA model discussed throughout the rest of this manuscript is the one we found best to characterize the major and minor features of the dataset.

The Immune and TAMs section had the largest distribution of documents, this was expected because the searching and screening phases of the scoping review targeted documents that discussed macrophage polarization in the context of cancer immunotherapy. Interestingly, the smallest topics were Biologics Delivery and Nanoparticles.

The most salient terms, the most frequent terms in the corpus, are shown in (Fig 4B). The bar length represents the term frequency in the entire dataset. The high prevalence of TAM is unsurprising, as the scoping review targeted studies in which nanoparticles interacted with TAMs.

### Identification of distinct categories within the dataset

**Fig 5:**
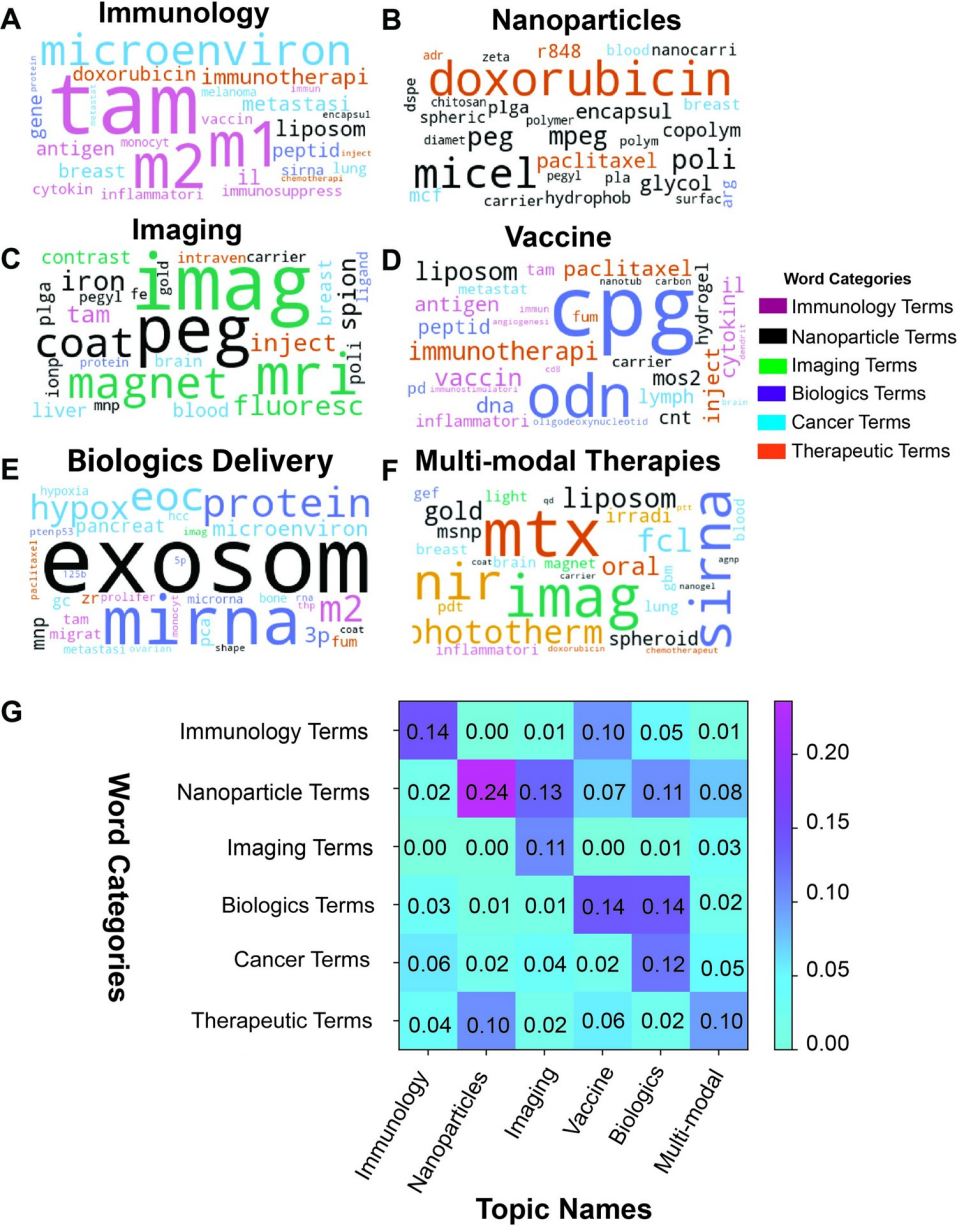
Dataset by word categories and topic names. [a] Word clouds by topic. Word size was determined by the frequency within the dataset while the colors were determined by their expert-assigned category. [b] Heat map of word categories within each topic by weight, determined by frequency within each topic.

To evaluate the topic contents, we manually categorized the most frequent words within each topic. Each article was assigned into a topic if the weight for that topic was at least 0.40. These words were then categorized by the authors into themes and sorted by frequency within the dataset. **(Fig 5)**. Six categories of words were manually identified: immunology, nanoparticles, imaging, biologics, cancer, and therapeutics. It should be noted that the categories are expert interpretations of the word. In the case of abbreviations and terms that are used across disciplines, there may be discrepancies in terms of placement. Nonetheless, the overall grouping of terms, fit well with the algorithm defined topics.

Some topics were largely dominated by one type of word. The Immunology topic contained terms such as TAM, M1, and M2 listed prominently. Nanoparticle topics, which also had the highest weighted score of all the topics, predominantly contained words associated with nanoparticle formulation and design. However, the other topics were identified by the unique combination of two or more categories. The Imaging topic by contrast, was highlighted by “imaging” and “nanoparticle” terms. The Biologics Delivery group was highlighted by “nanoparticle”, “nucleic acid” and “cancer” terms.

Furthermore, these trends were quantitatively validated by the weight scores (Fig 5B) The weight scores were calculated by dividing the frequency of words belonging to a given category by the total frequency of words in that topic. For example, in documents that were assigned to the nanoparticle topic, among all appearances of the 100 most frequent words, nanoparticle terms occurred approximately 24% of the time. It is important to note that words that were too vague to elucidate their meaning in the context of the documents were not included in this analysis, even if they did appear in the 100 most frequent terms. These include words like “activ”, “method”, and “cancer”. Generally, there was high correlation between a word category and corresponding topic (Fig 5B). However, the map also demonstrates the similarities and overlap between specific topics. The Vaccine and Biologics Delivery topics both had a higher weight for biologics terms. Similarly, the Nanoparticle and Multi-Modal Therapy topics had higher weights for nanoparticle and therapeutic terms.

It should also be noted that while more than one topic contained nanoparticles as a predominant word category, the kinds of nanoparticle terms that appeared in each topic were unique. For instance, the nanoparticle terms in the imaging topic such as “iron” and “gold” did not appear in the Nanoparticle topic (i.e., PLGA, micelle, chitosan) (Fig 4A). The biologics word category with respect to the Vaccine topic contained “cpg”, “odn”, and “antigen” in comparison to the Biologics Delivery group which contained “mRNA”, “microRNA”, and “3p”.

We also noticed that organs were more prominent based on the topic. For example, breast, liver, brain, and blood were key terms that appeared in the Imaging topic. In Biologics Delivery, pancreas, bone, prostate, and hepatocellular organs were prevalent.

#### Immunology

Papers in the Immunology topic tend to prioritize TAM repolarization. These papers describe the TAM phenotype and strategies to convert them into anti-tumoral pro-inflammatory macrophages. These strategies include cytokine treatment, microRNA gene delivery, and small molecule stimulation of pro-inflammatory transcriptional pathways such as NF-kB and TLR 7/8. Notably, these therapeutic cargoes are distinct from the types of therapeutics seen in the Nanoparticle Topic. Rather than attempting to induce death in cancer cells, these strategies target TAM function, migration [26, 27], transcriptional activity, and antigen presentation [28]. This topic was particularly strong within the data set, and consistently appeared during model reruns.

#### Nanoparticles

The nanoparticle topic showcases papers with a strong focus on nanoparticle characterization and development for anti-tumor therapy. While nanoparticles appear in other topics, papers that describe *in vivo* pharmacokinetic/pharmacodynamic [PK/PD], *in vitro* and *in vivo* cytotoxicity dose measurements, and structural and loading characterization are most likely to be found in this topic. Polymeric nanoparticles were the most common compositional type found followed by lipid-based nanoparticles. The polymers terms with the highest word frequency (Fig 5B) were PLGA (poly[lactic-co-glycolic acid]), PEG (polyethylene glycol), PLA (polylactic acid), and chitosan (a biopolymer of polysaccharides). In addition, chemotherapeutic drugs such as Paclitaxel, Docetaxel, and Doxorubicin feature prominently in this section. Such papers often intended to overcome multidrug resistance [29–31]. This was another strong topic, persistently appearing alongside the immunology topic when the model was rerun.

#### Imaging

The imaging topic contains papers that use nanoparticles to visualize macrophages within tumors. The most prominent nanoparticles are metallic nanoparticles that provide great contrast in MRI imaging. Given the high prevalence of macrophages within solid tumors, these nanoparticles are also used to detect tumor metastasis [32–34]. Nanoparticles are conjugated with mannose [35–37] to enhance uptake in monocyte, macrophage, and dendritic cell populations. Moreover, these nanoparticles are used as theranostics (diagnostics + therapeutics). Many papers combine metal particles with another therapeutic:curcumin [38] and a COX-2 inhibitor [39] are two such examples. This topic has some overlap to the Multi-modal Therapy Topic.

*Biologics delivery*. The Biologics Delivery Topic featured papers that used a variety of methods to deliver biologics (e.g., proteins and nucleic acids). Extracellular vesicles [EV] drug delivery was particularly common within this topic. Extracellular vesicles, which include exosomes, are released by cells, and can carry a wide array of cell constituents like DNA, RNA, and proteins [40]. EVs can be used as therapeutics, either as drug delivery vehicles or as the active therapeutic. In these papers, studies used EVs to deliver nucleic acids, such as microRNA, but proteins like an anti-CD137 antibody and SIRPα were delivered using nanoparticles [41, 42]. Besides extracellular vesicles, this category also included nanoparticles with different surface functionalizations such as macrophage membrane, cancer cell membrane, and CD47. These functionalizations improved tumor targeting by avoiding macrophage uptake in the circulation [43–45].

#### Vaccines

As the title describes, the topic contains vaccine strategies for macrophage reprogramming. Here, cargo strategies were not primarily chemotherapeutics but antigens and adjuvants designed to initiate pro-inflammatory activation. Lymph nodes were featured in this topic because of the organ’s significance in adaptive immunity and as a site of metastasis. While this topic did include more traditional vaccines against different cancer types like melanoma [46], it also encompassed therapies that aimed at activating the immune system, like modulating the lymphocyte populations within the tumor [47], reprogramming the tumor microenvironment [48], or heightening the patients’ immune response [49]. CpG ODNs were commonly seen in these studies as an immune stimulator and adjuvant. We observed that liposomes appeared in high frequency in this category, suggesting a potential preference of lipid formulations for vaccines (Fig 5D). Notably, the cancer vaccine field is much larger than what is represented in the current dataset because of the exclusion of vaccines in search string design.

#### Multi-modal therapies

The Multi-modal Therapies Topic encapsulated papers that employ more than one strategy. Photothermal Therapy, Photodynamic Therapy, or magnetic fields were combined with chemotherapy or immunotherapy. Interestingly, we found glioblastoma to be a prevalent cancer type in this category. These studies often utilized the coactive relationship between nanoparticles and the second strategy, like how metal nanoparticles will be rapidly heated during photothermal therapy, allowing targeted heat to the tumor and minimizing off-target effect [50–52]. Another second strategy seen was an alternative delivery method, like inhalation [53, 54]. As stated previously, there was overlap into the imaging category, with the second strategy being the imaging component, assisted by the nanoparticles. Some overlapping studies included nanoparticles being used as radiosensitizers [55], where nanoparticles enhance a tumor’s sensitivity to radiation through reactive oxygen species production [56]. Overall, this topic’s studies focused on how nanoparticles and a second strategy or therapy can amplify each other’s effects, resulting in a synergistic relationship.

#### Method to implement a living scoping review

**Fig 6:**
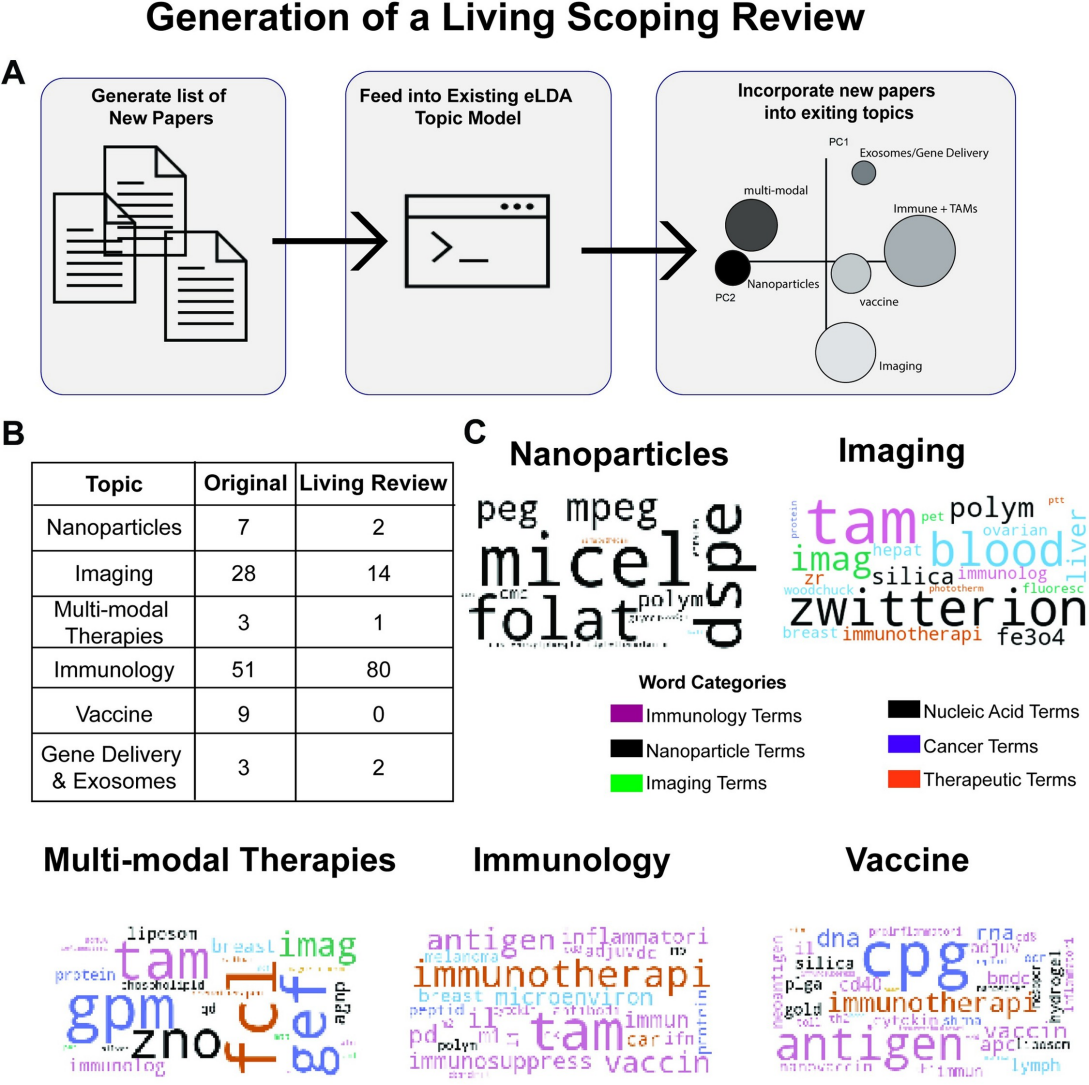
Living scoping review. [a] Schematic of process used to generate a living scoping review. [b] Table comparing the percentages of papers that were distributed into each topic between the original dataset and the newly acquired papers that form the Living Review. [c] Word cloud of most significant word by topic of papers included in the Living Review. Word size was determined by the frequency within the dataset while the colors were determined by their category.

While the topic model provided a great assessment of the existing literature, this review will eventually become outdated and lose its utility. Thus, we sought to determine whether we could use the eLDA topic model to generate a living scoping review, a system that could be continuously updated **(Fig 6A)** [17, 18, 57]. We used the eLDA topic model to extract topics from 95 abstracts that passed the full-text screening. The model identified five of the six topics in this new dataset (Fig 6B). The only topic that was not extracted was the Biologics Delivery topic. This is unsurprising since this topic was the smallest in the original dataset as visualized via the intertopic distance map (Fig 4). We followed the same methodology to identify topic trends using wordclouds (Fig 6C). We found a large degree of consistency between frequent words in the original and the newer topics. For example, the Immunology topic still contained words related to “TAMs” and ‘immunotherapy”. Additionally, the Nanoparticles topic still had a focus on polymeric nanoparticle terms such as “Micelle”, “DSPE”, and “PEG”.

## Conclusion

We used machine learning enabled topic modeling to categorize pre-clinical literature regarding macrophage targeting nanoparticle literature within the cancer field. By using the eLDA model, we were able to analyze a huge subset of data collected during our scoping review. To systematically retrieve data from over 800 papers by hand is a huge undertaking and would have required an unrealistic time investment.

The eLDA topic modeling analysis revealed important insights on the last 20 years of macrophage-targeted cancer nanotherapeutics. Based on word frequency, six distinct topics emerged and were labeled as “Immunology, Nanoparticles, Imaging, Biologics Delivery, Vaccines, and Multi-modal Therapies.” The topics demonstrated different themes within the field. The Immunology topic highlighted the TAM repolarization strategy, where researchers attempt to make the tumor this microenvironment less immunosuppressive by changing macrophage phenotype. As demonstrated in the topic’s word cloud, these papers often used liposomes and targeted breast cancer, lung cancer, or melanoma. The Nanoparticle topic focused on studies whose analysis was centered on nanoparticle formulation with detailed information about the formulation’s PD/PK and structure. The nanoparticles most often studied in this fashion were polymeric and were often loaded with chemotherapeutics. The Imaging topic demonstrated the strategy of using TAM’s phagocytic ability to uptake nanoparticles, often metal, to aid in imaging tumors. The most common targets for these imaging studies were shown to be tumors in the breast, liver, and brain. The Biologics Delivery topic very clearly illustrated the link between using exosomes or other vesicle-like particles for gene delivery, particularly miRNA. The Vaccine topic encompassed “traditional” vaccines where nanoparticles deliver an antigen often alongside an adjuvant. Studies also aimed at activating the immune system to encourage an immune response to the tumor. CpG is very prominent within this category, expectedly, as it is a strong innate cell activator and a vaccine adjuvant. The Multi-Modal topic highlighted how some studies combine approaches for a synergistic treatment strategy, with NIR [near infrared radiation], photothermal therapy, and imaging being augmented by the administered nanoparticle.

While each topic employed nanoparticles, the eLDA model was able to capture distinct nanoparticle types and therapeutic features, demonstrating the scope of the TAM-targeted nanoparticle field. The word frequency tables generated using the eLDA model also allowed visualization of different cancer subtypes and organs associated with each topic as outlined above. This insight is invaluable for future therapeutic design and development, as well as an ability to draw connections between nanoparticle strategy and targeted cancer.

This study demonstrates how using topic modeling allows scientists to analyze large amounts of literature in a manner that can reduce prestige, journal, and geographical bias. However, as with all uses of machine learning, care must be given to the initial prompt used when generating the model to achieve the most accurate results. It is also recommended to collaborate with information specialists who have expertise in evidence synthesis methods when compiling search strategies to ensure comprehensive results.

It is important to consider that the topic modeling approach is a way to extract latent themes from a dataset. Data can often be categorized in multiple different ways, which is reflected in the fact that two topic models that are created using the same parameters and data can produce different results. In addition, the because the topics are associated with word frequency, it is dependent on the using the most relevant terms in their abstracts. The topics are representative of both individual researchers writing style preferences and scientific research content. This topic model should be interpreted as one way to organize and identify emerging themes within a large amount of information.

Another feature of this study is that topic modeling is a high-level overview of a set of studies rather than in-depth analysis. Topic modeling is best used as a tool to supplement a systematic review rather than replace the type of in-depth analysis achieved by manually extracting data through systematic evidence synthesis. Using topic modeling as a tool for literature review is not without precedent, as topic models have been used to analyze literature on various research areas, including nursing research, cancer immunotherapy, and emergency medicine research, among others [5–7, 58]. This topic modeling review differs from similar ones in the field in that the topics were manually reviewed to ensure they accurately represented trends within the field. This type of high-level analysis could help identify possible topics for a more extensive systematic review.

The original dataset was rigorously vetted through a two-step title/abstract followed by full-text multi-reviewer screening to ensure the adherence to the TAM nanoparticle theme. With the model now established, we generated a living scoping review that could classify new literature that was not included in the original dataset, expanding the utility of a topic model beyond what has been previously shown. We envision that topic modeling approaches can be adopted by an individual or a team of investigators for keeping up-to-date with current literature by sorting new papers into existing topics. In addition, these models could be used to curate existing documents stored in reference managers. Topic modeling is a useful tool that can also be used to assess new scientific fields quickly and thoroughly.

## Supporting information

S1 Checklist (DOCX)

S1 File Search strategies used to find relevant studies for screening. This includes search strategies for PubMed [Medline], Web of Science Core Collection [Clarivate], Scopus [Elsevier], IEEE Xplore, Biotechnology & BioEngineering Abstracts [ProQuest], and Google Scholar.
(DOCX)

S2 File Dataset used to construct eLDA topic model. This dataset includes the title, journal, authors, and abstract of the included studies.
(XLSX)

S3 File Code submission. The purpose of this notebook is to provide example code that was used for the creation of this paper.
(IPYNB)
